# RNA methylation in neurodevelopment and related diseases

**DOI:** 10.3724/abbs.2024159

**Published:** 2024-09-27

**Authors:** Wenjuan Xia, Yue Liu, Jiafeng Lu, Hoi-Hung Cheung, Qingxia Meng, Boxian Huang

**Affiliations:** 1 State Key Laboratory of Reproductive Medicine and Offspring Health (Suzhou) Suzhou Affiliated Hospital of Nanjing Medical University Suzhou Municipal Hospital Gusu School Nanjing Medical University Suzhou 215002 China; 2 School of Biomedical Sciences Faculty of Medicine The Chinese University of Hong Kong Hong Kong 999077 China

**Keywords:** RNA methylation, neurodevelopment, N6-methyladenine (m6A), 5-methylcytidine (m5C)

## Abstract

Biological development and genetic information transfer are governed by genetic, epigenetic, transcriptional, and posttranscriptional mechanisms. RNA methylation, the attachment of methyl (–CH _3_) groups to RNA molecules, is a posttranscriptional modification that has gained increasing attention in recent years because of its role in RNA epitranscriptomics. RNA modifications (RMs) influence various aspects of RNA metabolism and are involved in the regulation of diverse biological processes and diseases. Neural cell types emerge at specific stages of brain development, and recent studies have revealed that neurodevelopment, aging, and disease are tightly linked to transcriptome dysregulation. In this review, we discuss the roles of N6-methyladenine (m6A) and 5-methylcytidine (m5C) RNA modifications in neurodevelopment, physiological functions, and related diseases.

## Introduction

Diverse and dynamic gene regulation by RNA modifications is critical for defining cellular function and phenotype. Among the more than 170 different chemical modifications in eukaryotic cells, the ones that have been intensively researched in the epitranscriptome are N6-methyladenosine (m6A), N6,2′-O-dimethyladenosine (m6Am), N1-methyladenosine (m1A), 5-methylcytosine (m5C), 5-hydroxymethylcytosine (hm5C), and pseudouridine (ψ) [1]. These RNA modifications impact RNA metabolism by regulating RNA processing events such as alternative splicing, transportation, stability, translation, and miRNA processing [ 2– 4]. Recent advancements in high-throughput sequencing technologies have enabled transcriptome-wide mapping of these modifications, revealing their pervasive and dynamic presence across different biological situations.

The central nervous system (CNS), which includes the brain and spinal cord, is the primary control center for coordinating information processing and responses in organisms. The formation and function of the CNS are regulated by a myriad of factors, among which RNA modifications have emerged as major contributors [ 5, 6]. m6A is the most prevalent RNA modification in the mammalian transcriptome [7]. It has several effects on the nervous system, including self-renewal of neural stem cells, cognitive processes such as learning and memory, maturation of neurons, expansion of synaptic connections, and proliferation of glioma cells [ 8, 9]. m5C modifications are particularly common in brain tissues and play a significant role in the CNS [10]. For example, m5C in neurons has been associated with the cellular stress response and programmed cell death [11]. This study revealed a strong correlation between m5C modifications and both brain angiogenesis and nervous system development in zebrafish brains exposed to hypoxic circumstances [1].

These findings indicate that both m5C and m6A modifications play significant roles in brain development and function, as well as in the initiation of neurological diseases. However, for a more comprehensive understanding of these relationships and their potential therapeutic applications, additional research is needed. This review aims to provide a thorough and extensive summary of our current understanding of RNA modifications, with a specific focus on m6A and m5C. We focus on their distribution, regulation, and functional roles in mammalian neurodevelopment, physiology, and disease. Additionally, we address the challenges faced in the study of RNA modifications and consider potential future paths in this field.

## Regulators of m6A and m5C

### Regulators of m6A

m6A is the most characteristic modification, accounting for approximately 0.1%–0.4% of the mammalian genome. m6A sites can be detected in the consensus sequence RRACH (where R represents purine bases A or G, and H corresponds to A, C, or U). The effects of m6A on mRNAs are mediated by a series of progressively discovered m6A readers, m6A writers, and erasers.

#### m6A writers

The methyl group can be written on the adenosine base at the nitrogen-6 position of the target mRNA by the methyltransferase METTL3/14. This process occurs in conjunction with RBM15/15B, ZC3H13, VIRMA and WTAP at the same time.

#### m6A erasers

The demethylases FTO and ALKBH5 can dynamically regulate m6A modifications.

#### m6A readers

Two m6A-dependent RNA binding modes have been identified for reader proteins: direct and indirect. The direct mode is mediated by dedicated RNA binding domains within reader proteins, such as YTHDF1/2/3, YTHDC1/2, and IGF2BP1/2/3. On the other hand, indirect RNA binding relies on the thermodynamically unstable double helix structure of m6A, where methylation influences the RNA structure, exposing the protein binding motif known as the “m6A switch”. Proteins such as HNRNPA2B1 can subsequently bind to exercise recognition and mediate m6A function in a specific context. There are also two m6A readers, G3BP1 and G3BP2, that preferentially bind to unmethylated sequences [ 12, 13 ].

### Regulators of m5C

The modification of 5-methylcytidine (m5C) is most common in transfer RNA (tRNA) and ribosomal RNA (rRNA). With advancements in technology, messenger RNA (mRNA) and long noncoding RNA (lncRNA) have gradually been identified. m5C modification is catalyzed by methyltransferases, and S-adenosyl-L-methionine is used as a donor to transfer methyl groups to RNA.

#### m5C writers

The core complex of m5C methyltransferases consists of TRDMT1 and the NOL1/NSUN protein family, which includes seven members: NSUN1 to NSUN7 and NSUN5a/b/c [14]. The most specific writer is NSUN2 (TRM4).

#### m5C erasers

m5C modification is reversible by the demethylase TET family (TET1/2/3) and ALKBH1. In DNA, TET proteins can catalyze the oxidation of m5C to form 5-hydroxymethylcytosine (5hmC), 5-formylcytosine (5fC), and 5-carboxylcytosine (5caC) [15]. In RNA, 5-methylcytidine is used to refer to m5C [16]. TET-mediated RNA m5C oxidation has been shown to regulate mRNA stability, translation efficiency, and splicing.

#### m5C readers

m5C can be recognized by the “readers” ALYREF and YBX1. ALYREF is the first reader identified in the nucleus, and YBX1 also stabilizes m5C-labeled RNA [ 7, 17]. TET proteins also interact with ALYREF to modulate their binding affinity and subsequent effects [18].

## Spatial and Temporal Dynamics of RNA Methylation in the Nervous System

**Figure 1:**
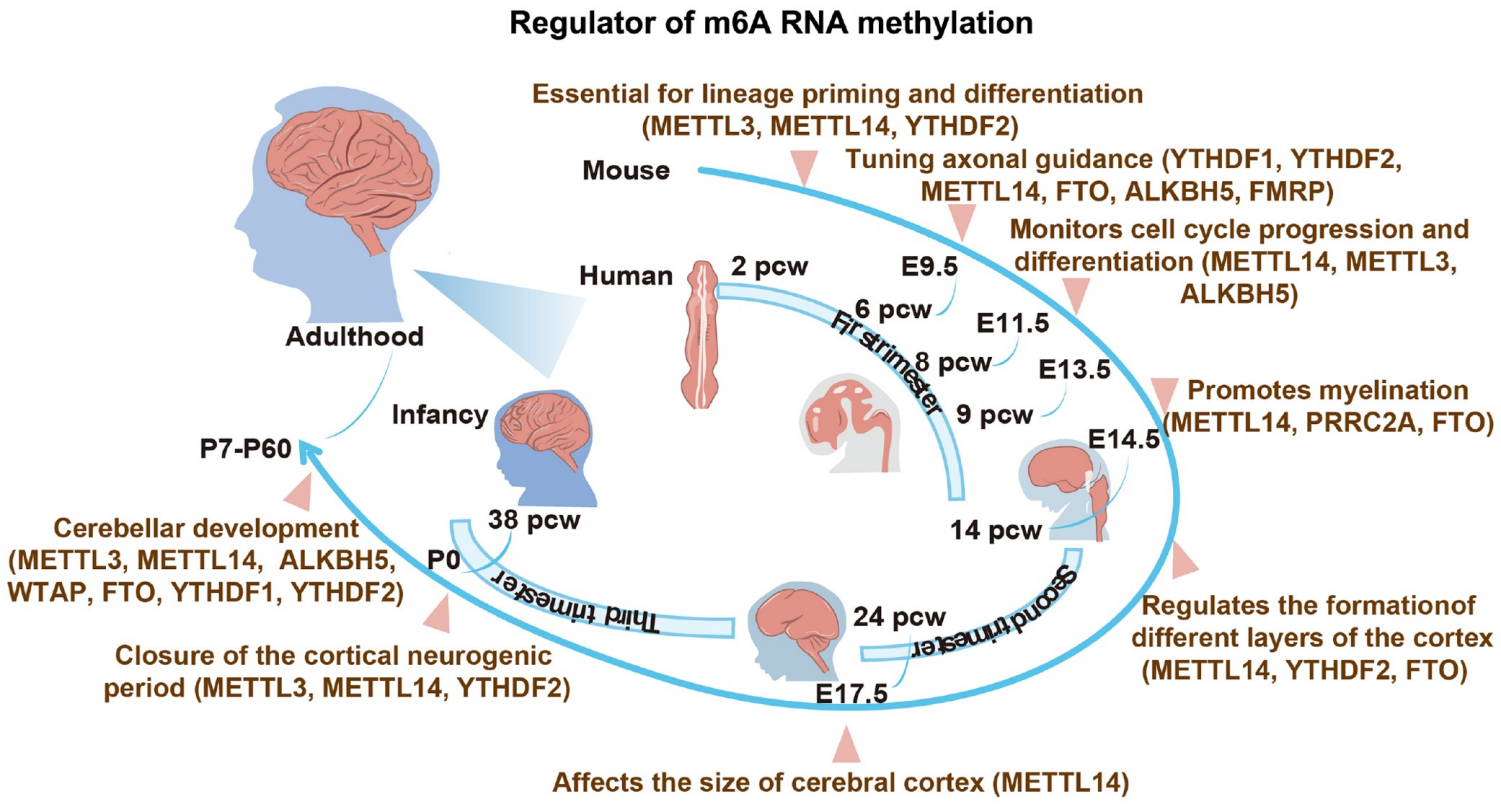
Figure 1 Regulatory effects of m6A regulators during the brain development process in humans and mice m6A plays a regulatory role in brain development, impacting the formation of the cerebral cortex and cerebellar neurodevelopment during the three trimesters of embryonic development and postnatal stages.

The coordination of typical brain development is a process that occurs in both space and time, necessitating the activation of accurate signals at appropriate time intervals. These signals guide the migration of different types of cells, both neural and nonneural, to their specific layers. Transcriptional regulation is crucial in this development, as various RNA modifications and transcription factors may control important activities. These processes involve prompt termination of the mitotic cycle, shedding of progenitor stemness, and acquisition of neuronal properties [17], as shown in Figure 1.

Transcriptome-wide m6A sequencing has demonstrated the widespread occurrence of m6A modifications in many human and mouse tissues. The highest expression of this modification was identified in the brain. This study revealed dynamic spatial and temporal features throughout both embryonic and postnatal neurogenesis. Specifically, m6A is expressed in neural progenitor cells (NPCs) and radial glial cells (RGCs), also known as neural stem cells (NSCs), in embryonic forebrain organoids [19]. During postnatal neural development, m6A is enriched in certain regions of the brain, such as the spinal cord, dorsal root ganglia, cerebellum, hypothalamus, hippocampus, and cortex. Compared with glial cells, neurons in the cerebral cortex and cerebellum of mice exhibit higher m6A levels [ 19, 20]. Over time, the number of m6A sites in the mouse brain significantly increases from adolescence to old age, which is similar to the findings in humans [21]. In terms of genomic distribution, m6A peaks are commonly located in the coding sequence (CDS), 3′UTR, and termination codon regions. In the mouse cortex, m6A-specific peaks are primarily concentrated in the CDS, whereas in the cerebellum, specific peaks are distributed in the initiation codon and 3′UTR [20]. In the cerebellum of postnatal mice, m6A peaks are particularly abundant in the CDS and stop codon regions at P7, a time when cells are proliferating and immature. However, at P60, when neurons are well differentiated and mature, these peaks are concentrated in the initiation codon region [22]. These findings indicate that the patterns of m6A deposition onto RNA undergo significant changes in tandem with the progression of cerebellar development.

m5C is widespread in brain tissue, but its identification is constrained by existing techniques. For example, over 7000 m5C sites have been detected in mouse embryonic stem cells [23]. However, RNA BS-seq identified a total of 501 m5C sites with high confidence in NSCs, neurons, and mouse brains at postnatal day 0 (P0), P17, and 6 weeks [24]. m5C levels exhibit a dynamic pattern during early postnatal brain development. It decreases from P0 to P17 and remains relatively stable from P17 to 6 weeks without any noteworthy changes [24]. These findings suggest that m5C modification may affect the development of neural circuits at P17 [25]. NSUN2 and ALYREF are more abundant in NSCs, but TET1 and TET3 are more highly expressed in neurons [24]. NSUN2, a m5C writer, is expressed in the early neuroectodermal cells of 6-week-old human embryos. These cells are capable of differentiating into distinct types of neurons and glial cells that are specific to different regions. Mice lacking both the Nsun2 and Dnmt2 genes exhibit a decrease in the thickness and organization of the cerebral cortex [ 26, 27]. The enrichment and dynamic expression of m6A and m5C in brain tissues suggest that RNA methylation can regulate nervous system development and influence physiological disorders.

## Regulation of RNA Modification during Neurodevelopment

Brain development during the fetal period involves the processes of neuron generation, migration and differentiation. The development of the mammalian central nervous system begins during early embryogenesis with the formation of a neural plate composed of neural ectoderm. m6A and m5C play important roles in neurodevelopment, such as neurogenesis, cerebellar development, synapse formation, and the functioning of the hypothalamic-pituitary-gonadal (HPG) axis.

### Cortical neurogenesis

**Figure 2:**
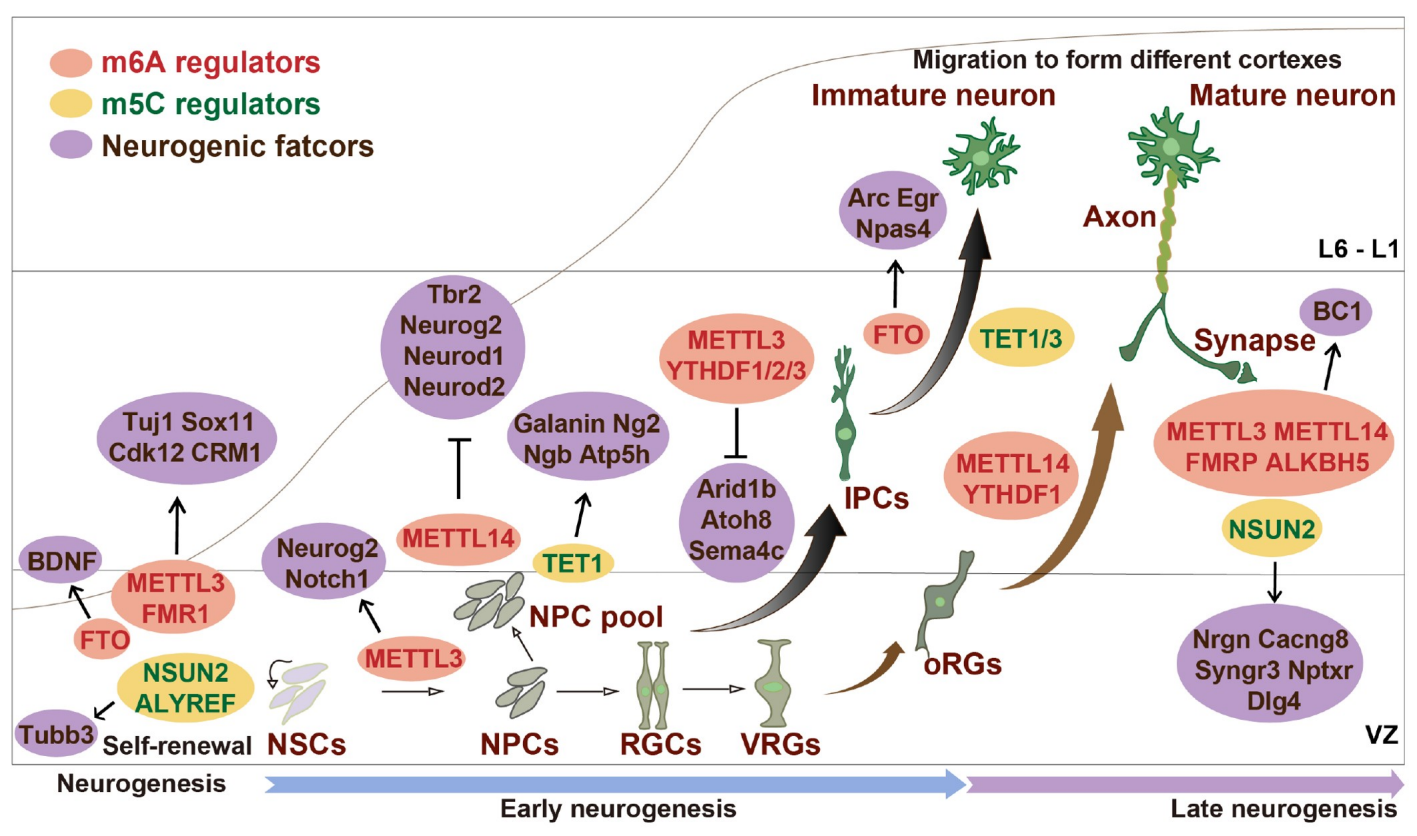
Figure 2 Modifications of RNA methylation influence neural differentiation Early neurogenesis is regulated mainly by m6A regulators such as METTL3, FMR1, and FTO, as well as m5C regulators such as NSUN2 and ALYREF, to control the self-renewal of neural stem cells. RGCs regulate IPC transformation mainly through m6A, such as METTL3, METTL14, and the YTH domain readers YTHDF1/2/3. In late neurogenesis, FMR1, FTO, TET1, and TET3 affect the differentiation of RGCs into neurons, and m6A is essential for axon extension and synaptic function. NSUN2 affects the formation and plasticity of synapses.

Neurogenesis refers to the proliferation of NSCs and their division into NPCs, which in turn generate nascent neurons and establish synaptic connections with other neurons [28]. This process begins in early embryonic development, persists throughout infancy and childhood, and ultimately results in the formation of mature neurons [29]. Cortical neurogenesis occurs in embryonic stages in the ventricular zone (VZ) and subventricular zone (SVZ). Neural crest cells form at embryonic day 8 (E8) in mice and give rise to proliferating RGCs at E14, which serve as progenitors for all neural cells [30]. The roles of regulators of m6A and m5C in neurogenes are shown in Figure 2 .

m6A is a crucial RNA modification that regulates lineage priming and differentiation. Previous studies have shown that m6A is required for the maintenance of multipotent neural stem cells and their differentiation into neurons during cortical development [ 19, 31]. Multiple m6A regulators are involved in neurogenesis during both the embryonic and postnatal stages. METTL14 expression is highest in RGCs, and its depletion results in an increase in the number of RGCs and intermediate progenitor cells (IPCs) and a prolonged cell cycle. This leads to delayed cortical neurogenesis and abnormalities in the production of neurons. A Nestin-Cre; Mettl14 ^f/f^ conditional knockout (cKO) mouse model revealed that the deletion of m6A in NPCs impairs forebrain development by altering the expressions of Tbr2, Neurog2, and Neurod1/2 at E17.5, resulting in a delay in the generation of neuron subtypes in different cortical layers [19]. Depletion of METTL3 can decrease the differentiation of newborn neurons in the E15.5 mouse cortex and disrupt the proliferation and cell cycle progression of adult neural stem cells (aNSCs) by influencing the activity of the histone methyltransferase Ezh2 [ 32 , 33]. Loss of m6A increases the number of NPCs and the expansion of IPCs but not RGCs. Additionally, it alters the morphology of neonatal neurons in the postnatal brain [32].

m6A reader proteins are responsible for brain development. Loss of TYHDF2 impairs embryonic development and delays the degradation of neural stem/progenitor cell (NSPC) differentiation [31]. Consistent with these findings, knocking out both *YTHDF1* and *YTHDF2* in mice results in a prolonged cell cycle in NPCs and a decrease in the number of productive neurons, similar to the findings in *METTL14*-cKO mice [34]. Depletion of YTHDF proteins upregulates the expressions of *Arid1b*, *Atoh8*, and *Sema4c*, which are associated with autism spectrum disorder (ASD). YTHDF2 plays a role in stabilizing mRNA, and we speculate that the METTL3-METTL14/YTHDF2 axis may be closely connected to neurogenesis, which deserves further investigation. Fragile X mental retardation protein (FMRP), encoded by *Fmr1*, is involved in the cell cycle of the NPC from early development until the postnatal stage [35]. In the mouse cerebral cortex, FMRP target mRNAs are highly enriched in m6A modifications [36].

The m6A methyltransferase FTO is significantly expressed during postnatal neurogenesis, and its absence leads to alterations in brain-derived neurotrophic factor pathways, specifically BDNF, which results in the apoptosis of newborn neurons. Additionally, it diminishes the proliferation and differentiation of aNSCs *in vivo* [ 37, 38]. These findings further support the role of m6A in brain development and cellular differentiation.

In addition to the regulation of neurogenesis by m6A, m5C is also present in nerve cells, and its regulators play important roles in neurogenesis [26]. As mentioned earlier, m5C sites have been detected in mouse neural stem cells, neurons, and the whole brain [23]. However, additional research is needed to determine the earliest moment at which they initially manifest in embryonic neural stem cells. In mice, the absence of m5C-modified tRNA fragments leads to impaired neural stem cell migration, differentiation, and brain development [26]. The primary methyltransferase responsible for the formation of m5C is NSUN2, which is highly expressed in the NPCs of the developing human brain and gradually decreases during stem cell differentiation. Nsun2-deficient mice exhibit a reduction in upper-layer neurons in the cerebral cortex [39], along with poor glutamatergic neurotransmission and synaptic plasticity [40]. NSUN2 deficiency leads to microcephaly by reducing the population of upper cortical neurons [41]. NSUN2 also regulates the nucleoplasmic shuttling of ALYREF and specifically recognizes m5C [42]. Further investigations are needed to determine whether NSUN2 synergistically regulates brain development through dynamic interactions with ALYREF. Knockout of *DNMT2*, another m5C writer, in zebrafish embryos results in abnormalities in hypothalamic and mesencephalic neurogenesis [43].

Recent studies have revealed the function of 5-methylcytosine-demethylation in neurogenesis. The erasers that activate the DNA demethylation of 5mC to 5hmC are TET1/2/3, whereas TET1 and TET2 activate the demethylation of m5C to hm5C in RNAs [44]. Specific deletion of TET1 via nestin-Cre in adult mice downregulates the expressions of genes related to NPC proliferation, such as *galanin* and *ng2*, resulting in a reduction in the number of NPCs in the SGZ region and impairing adult neurogenesis [45]. Whether m5C demethylation affects neurogenesis through RNA or DNA requires further study.

The Neurod family is essential for the development of the brain cortex. Transcription factors such as *neurog1* and *neurog2* influence the differentiation and maturation of neurons. Additionally, various signaling pathways are important for neurogenesis, including the activation of the Notch pathway, which may affect the expression or secretion of BDNF. BDNF, in turn, can influence the expressions of *Neurod* genes by activating its receptor, *TrkB*. Moreover, the expressions of *Neurod* genes may affect components of Notch signaling, thereby influencing the fate of neural stem cells.

RNA modifications such as m6A and m5C can regulate the expressions of genes related to neurogenesis by affecting the stability and translation of RNA, which impacts the proliferation of NSCs and the differentiation and maturation of neurons. m6A modifications primarily occur in the 3′UTR and CDS of mRNAs, whereas m5C modifications are concentrated in the 5′UTR and CDS. m6A is associated with RNA splicing, export, translation, and degradation processes, whereas m5C is related to RNA stability and structure. The precise coregulatory patterns of these modifications during neurogenesis remain incompletely known.

### Cerebellar development

Cerebellar development closely parallels brain development, since it adheres faithfully to the sequential stages of early NSC generation, NPC proliferation, migration, differentiation, and synapse formation. Granule neurons are the primary cell type found in the cerebellum of mammals and account for more than half of the total number of neurons in the brain. Structurally, the composition of this entity can be categorized into three distinct layers: the molecular layer (ML), Purkinje cell layer (PCL), and granule cell layer (GCL).

Cerebellar development predominantly occurs postnatally and is characterized by increased m6A levels relative to those in the cerebral cortex. The expressions of METTL3, METTL14, WTAP, ALKBH5 and FTO progressively decrease in cells of the inner granular layer (IGL) of the cerebellum during development but simultaneously increase in Purkinje cells (PCs) [22]. METTL3 ^cko^ leads to a reduction in the number of granule cells (GCs) in the external granule layer (EGL) of the cerebellum, resulting in cerebellar hypoplasia [46]. In contrast, METTL3 expression increases during the differentiation of granulosa cell precursor cells (GCPs). Deletion of METTL3 does not affect the proliferation of GCPs but does increase the apoptosis of nascent GCs. Specific knockout of *WTAP*, a member of the m6A methyltransferase complex in PCs, leads to cerebellar atrophy [47]. Additionally, the demethylase ALKBH5 gradually increases during physiological aging in mice [48]. ALKBH5 dysregulation leads to an aberrant m6A pattern in mRNA, leading to developmental defects in the cerebellum [22]. These findings suggest that maintaining a balance of mRNA methylation is critical for proper cerebellar development.

The exploration of the role of m5C modifications on RNA in cerebellar development has been limited, but DNA modifications have received increasing attention. For example, 5mC, DNA methyltransferase 1 (DNMT1) and DNA methyltransferase 3a (DNMT3a) are highly expressed in NPCs or granule cells in the EGL and IGL, indicating that 5mC-mediated DNA methylation is crucial for the differentiation of cerebellar granule cells [49].

### Regulation of synaptic function

m6A methylation is important for regulating synaptic function and axonal guidance. Adult METTL14-deficient mice exhibit reduced expressions of genes enriched at synapses, postsynaptic membranes, axons, and dendrites [50]. Knockdown of *Mettl14* results in PTEN deficiency and reduced axonal regeneration in retinal ganglion neurons [51]. METTLE3-null rats exhibit atypical synaptic transmission [52]. The reader YTHDF1 regulates the translation level of Robo3.1, a receptor involved in guiding spinal axons across the midline without affecting its mRNA levels, and YTHDF1 ^cKO^ causes anterior crossed axon guidance defects and impaired synaptic transmission [ 53, 54]. *YTHDF1* and *YTHDF3* knockdown in mouse hippocampal neurons resulted in decreased m6A levels, defects in excitatory synaptic structure and function, clusters of postsynaptic density-95 (PSD-95), altered expression of the AMPA receptor subunit GluA1, and reduced excitatory postsynaptic transmission, in addition to a narrowed spinal head phenotype [55]. FMRP colocalizes with m6A-modified RNAs at postsynaptic sites and affects the short-term plasticity of glutamatergic synapses. FMRP suppresses the process of translation of dendritic mRNAs encoding the cytoskeletal proteins Arc/Arg3.1 and MAP1B, as well as the kinase α-CaMKII [36]. Demethylase is also involved in regulating synaptic plasticity and axonal extension. FTO is significantly enriched in axons, and the loss of FTO can impede axon extension by reducing the translation of GAP-43 [56]. FTO-deficient animals have elevated methylation levels of mRNA, which is linked to synaptic transmission and impaired dopamine release in the striatum and midbrain [57]. Synaptic plasticity requires mRNA to be transcribed in the nucleus. ALKBH5-mediated demethylation, which mostly occurs in the nucleus, occurs mainly at synapses during short-term plasticity as well as within synaptic ribosomes [ 53, 58].

With the specific knockout of *NSUN2* in mouse prefrontal cortex neurons, the m5C level in tRNA is significantly decreased, resulting in altered 1/4 protein expression and causing synaptic signaling impairment and behavioral disorders [39]. Synaptic plasticity in the hippocampal NMDAR-LTP system is impaired in the NSUN2-deficient murine model [59]. However, it is still unclear whether this is caused by the regulation of m5C methylation, and no previous study has attempted to characterize the effects of m5C methylation of mRNAs on synaptic function.

### Hypothalamic-pituitary-gonadal (HPG) axis

m6A modification may play a role in initiating and coordinating sexual maturation along the hypothalamic-pituitary-gonadal (HPG) axis, where immature female rats can induce GnRH secretion via Mn while increasing the level of FTO and inhibiting the m6A level of GABAA receptor mRNA. Puberty can be delayed by inhibiting FTO because it reduces the hypothalamic expression of the GABAAR protein [60]. In the chicken hippocampus, the clock genes in the hypothalamus and pituitary have a circadian rhythm of expression, and m6A levels in the hypothalamus similarly exhibit diurnal variations, increasing throughout the day and decreasing at night [61]. Studies on the regulatory properties of m6A in the HPG of Salmo salar have also been conducted, although they are still insufficiently explored and require further investigation.

m6A affects the expressions of histones, such as H3K27ac, H3K27me3, and H3K4me3, during neuronal development. Recent research has indicated that the transcription of endogenous retroviruses (ERVs) is activated in the aged neurons of Cynomolgus monkeys [62]. Like m6A, RNA methylation can promote the degradation of ERV transcripts, thereby maintaining genomic stability [63]. The dynamic expression of m6A in the mammalian brain suggests that it influences the ERV family, which in turn may contribute to neuronal differentiation or synaptic plasticity. Innate cellular immunity can be triggered, and genomic instability can be caused by ERV activation. Most ERVs require DNA methylation or histone modifications, such as H3K9me3 and H3K27ac, to remain silent and preserve cell homeostasis. To determine whether m6A synergistically regulates the development of the nervous system by affecting histone modifications on ERVs and DNA methylation, m6A regulator knockout mice should be established for further study.

## RNA Methylation Is Involved in the Regulation of Physiological Functions in the Brain

### Learning and behavior

Memory is related to the long-term formation of synaptic morphology and function, also known as synaptic plasticity. Memories initially originate in the hippocampus and gradually stabilize in the cortex [64]. Neural circuits and beginning memories in mice can be influenced by early life experiences [65]. The ability of the hippocampus to consolidate long-term memory is associated with METTL3 [66]. *METTL3* knockout in the hippocampus of rats leads to learning and memory deficits [52]. METTL14 deletion in two neurons (striatonigral and striatopallidal) in the striatum of adult mice results in a phenotype characterized by impaired behavior and learning. The initial stages of response learning and reversal learning are important for the establishment of long-term memory and the modulation of neuronal excitability [50]. YTHDF1 recognizes m6A on target mRNAs to accelerate protein synthesis, which contributes to learning and memory formation in the hippocampus of mice. However, YTHDF1 deletion impairs synaptic transmission in the hippocampus and negatively affects memory formation [53]. Recent research has revealed that the establishment of short-term memory relies on YTHDF-mediated mushroom bodies, which are central to associative learning. YTHDF2 regulates the elongation of mossy fibers in dentate gyrus (DG) granule cell axons by controlling the stability of target transcripts and, thus, proteins. Deletion of the *YTHDF2* gene specifically in DG cells leads to excessive proliferation of mossy fibers (MFs) and impairs MFCA3 excitatory synapse development and transmission in the hyaline layer [ 67, 68]. FTO expressed in the nucleus and dendrites. After situational fear conditioning stimulation, the expression of *Fto* was decreased in the dendritic spines of neurons in CA1 region of the mice dorsal hippocampus, indicating that FTO inhibits memory formation [69]. FTO and METTL3 can affect mRNA degradation as behavior accumulates in mice, and knocking down FTO in the mouse prefrontal cortex (MPFC) results in a phenotype in which fear memory is enhanced and consolidated [70].

### Stress response

The levels of m6A and m6Am in the brain can be altered in response to UV irradiation-induced DNA damage, heat shock, hypoxia, and oxidative stress. These modifications bind to regulatory proteins that act as epigenomic–transcriptomic markers and are involved in the response to acute stress. They inhibit neuronal transport and synaptic plasticity-associated mRNA expression [ 71, 72]. The regulation of the stress response is significantly influenced by alterations in RNA methylation in two specific areas of the mouse brain: hypomethylation in the medial prefrontal cortex (PFC) and hypermethylation in the basolateral and central amygdala (AMY). METTL3-dependent upregulation during heat shock and knockdown of the reading protein YTHDC1 can result in increased levels of stress resistance and key heat shock protein (HSP) chaperones [73]. WTAP is specifically regulated within the AMY under stress. In addition, FTO, ALKBH5, and YTHDC1 have distinct regulatory patterns specific to different regions [74]. m6A-labeled mRNAs contribute to the assembly of stress granules, and YTH-reading proteins are the basis for stress granule formation [75].

Mammals rely on their past encounters to learn, establish memories, and adjust their actions to their surroundings to survive. The effect of m6A on memory appears to be dose-dependent, with both increases and decreases in m6A levels leading to improvements or impairments in memory. Further investigation is needed to fully understand the mechanisms underlying this relationship.

## RNA Methylation and Neuropsychiatric Disorders

Dysregulation, in turn, is associated with neuropsychiatric, neurodevelopmental, and neurodegenerative disorders. Neurodevelopmental disorders, including attention deficit hyperactivity disorder (ADHD), autism, and intellectual disability, reflect changes in the development of brain function. Neuropsychiatric disorders affect brain function and include bipolar disorder, major depressive disorder (MDD), and schizophrenia. Neurodegenerative diseases, including Alzheimer’s disease (AD) and Parkinson’s disease (PD), are among the most extensively studied areas [76]. The regulation of neurobiological processes by RNA modifications, especially in brain development and neurodevelopmental disorders, is shown in Table 1 .

**Table:** **Table 1** Biological effects of regulators of m6A and m5C in neuropsychiatric disorders.

Disease	Regulator	Effect	Regulatory mechanism	Ref.
ASD	METTL3	METTL3 reduces hippocampal neuron apoptosis.	Regulating the MALAT1/SFRP2/Wnt/β-catenin axis.	[77]
YTHDC2	YTHDC2 is potential risk site.	‒	[78]
NSUN2	Synaptic plasticity is impaired in NSun2-deficiency.	NMDAR-LTP mis-regulation.	[59]
FXS	YTHDF1/2	Inhibition of YTHDF1 can reduce thesymptoms of mental retardation syndrome.	YTHDF1 and FMR1 competitively bind to the m6A site.	[79]
FMRP	The production of neurons slows down.	Decreased FMRP results in decreased nuclear output of m6A-labeled RNA.	[80]
MDD	FTO	FTO is downregulated in the hippocampus of patients with MDD and mouse models ameliorated chronic restraint stress induced depressive-like behaviors TCAs increased FTO expression.	FTO kd decreases the expression of ADRB2 and SIRT1 through c-MYC, and FTO modulates hippocanpal synatic plasticity by targeting CaMKII/CREB signaling pathway in hippocampus.	[37]
ALKBH5	Alkbh5 was strongly associated with MDD.	‒	[81]
METTL3	METTL3 reduces hippocampal neurogenesis, spatial memory decline, and depression-like behaviors.	Mettl3-mediated m6A modification of *Lrp2* mRNA promotes neurogenesis through m6A reader Ythdc2 *in vitro*.	[82]
NSUN2/TET2	NSUN2 kd produces an antidepressant phenotype. TET2 enhances 5hmC level and Epo expression.	Neuronal NSUN2-deficiency decreases m5C level of tRNA, resulting in a loss of Gly-rich proteins.	[ 39, 83]
AD	METTL3	METTL3 kd leads to memory deficits, extensive synaptic loss, neuronal death, and multiple cellular alterations.	Overexpression of METTL3 in neurons salvaged Aβ-induced synaptic damage and cognitive impairment.	[84]
METTLE ko leads to aberrant expression and distribution of crucial genes in RGCs.	Accumulation of METTL3 positively correlate with Tau protein in the postmortem human AD samples.	[32]
FTO	FTO is involved in insulin defect-related AD.	Targeting TSC1-mTOR-Tau signaling.	[85]
METTL3 was increased and FTO was downregulated in APP/PS1 transgenic mice.	Abnormal methylation levels of the m6A RNA of the AMPA, NMDA and SEMA genes that encode synaptic function.	[32]
hnRNPA2/B1	Hippocampus hnRNPA2/B1 levels are sensitive to changes in cholinergic tone.	Regulation of hnRNPA2/B1 levels by cholinergic activity that interferes with alternative splicing in targeted neurons.	[86]

### Neurodevelopmental disorders

ASD can be divided into three categories: autism, Asperger’s syndrome, and pervasive developmental disorder not otherwise specified (PDD-NOS). Fragile X syndrome is a prevalent genetic disorder caused by the deletion of the *Fmr1* gene in FMRP. FMR1 is a m6A reader, and a previous study suggested that *Fmr1* gene deletion can delay neuronal production [ 35, 80], thus revealing a link between mRNA modification and ASD. In addition, the YTHDC family has been shown to serve as a potential risk locus for autism disorders in the population [ 78, 79]. In the hippocampal tissues of mouse models of autism, METTL3 stabilizes MALAT1 expression by increasing m6A modification of the host gene. This leads to the methylation of *Sfrp2* and subsequently reduces SFRP2 expression by recruiting related transcription factors to the promoter region of *Sfrp2* and promoting the activation of Wnt/β-linked protein signaling. At this stage, METTL3 inhibits autism-like symptoms and the death of hippocampal neurons [77].

Studies have shown that autism spectrum disorder is characterized by impaired control of synaptic plasticity, which is dependent on protein production. The NMDAR system could be considered a potential therapeutic target for autism. A protein synthesis-dependent form of synaptic plasticity in the hippocampal NMDAR-LTP system was impaired in the NSUN2-deficient murine model [59]. NSUN2 can control the rate of protein synthesis through direct regulation of RNA methylation, and NSUN2-mediated m5C RNA methylation could be a promising treatment strategy for addressing the NMDAR system in individuals with autism spectrum disorders.

### Neuropsychiatric disorders

m6A RNA methylation is also associated with neuropsychiatric disorders, and certain genetic variants of the FTO SNP rs9939609 and the ALKBH5 SNP rs12936694 increase the risk of MDD and attention-deficit/hyperactivity disorder. FTO expression was found to be reduced in the hippocampal region of MDD patients as well as in mouse models of depression. FTO regulates m6A modifications on the adrenergic receptor beta 2 ( *ADRB2*) gene to stabilize its expression. Activation of ADRB2 rescues depression-like behavior and spinal loss phenotypes [87]. The SNP in ALKBH5 is associated with various clinical features of MDD, including anxiety, developmental delay, and cognitive dysfunction. Further studies demonstrated that ALKBH5 ^OE^ stabilizes the mood-associated transcript FAAH and promotes depressive behavior in mice. In contrast, circSTAG1 decreased the translocation of ALKBH5 to the nucleus, leading to an increase in m6A methylation, thereby attenuating astrocyte dysfunction and depressive-like behavior [81].

The overexpression of m5C in the prefrontal cortex can lead to depressive-like behavior by affecting synaptic transmission. Conversely, reducing the expression of NSUN2 can result in an antidepressant effect [39].

### Neurodegenerative disorders

Alzheimer’s disease (AD) and Parkinson’s disease (PD) are the most common neurodegenerative diseases. m6A is involved in the expressions of key genes in AD [ 21 , 88]. During aging in mice, there is a notable increase in m6A sites, as well as an increase in METTL3 and IGFBP2 and a decrease in FTO in the cerebral cortex and hippocampus in AD [ 84, 85, 89]. In humans, patients with decreased expression levels of both METTL3 and NDUFA10 are more likely to develop Alzheimer’s disease [90]. Inhibiting METTL3 can delay the progression of mouse AD [91]. When FTO in the hippocampus is downregulated, the BDNE signaling pathway is inhibited. This aligns with the process of cognitive dysfunction in AD patients [92]. The decrease in cholinergic tension characterized by the loss of acetylcholine (ACh) is one of the features of AD. HNRNPA2/B1 levels interact with ACh activity because HNRNPA2/B1 mediates the target gene in neuron-selective splicing and causes disease [86]. The primary pathological change in PD is the degenerative death of dopaminergic neurons in the midbrain substantia nigra, which causes a significant decrease in the striatal dopamine content and leads to disease. m6A reduction induces N-methyl-D-aspartate receptor 1 (NMDA) expression, which increases oxidative stress and Ca ^2+^ influx, leading to the apoptosis of dopaminergic neurons [93]. In elderly individuals, NSUN6, NSUN7, and ALYREF are significantly differentially expressed [94]. As a m5C writer, NSUN2 deficiency can contribute to the development of neurodegeneration and an increase in tau hyperphosphorylation in humans [40].

The significance of m6A and m5C in the diagnosis and monitoring of various tumor types has been demonstrated. Abnormal expression and regulation of RNA methylation in neurological diseases may be promising new targets or screening indicators for the treatment of neuropsychiatric disorders in the future. STM2457, a small molecule inhibitor of METTL3, has shown promise in the treatment of non-small cell lung cancer [95]. However, additional clinical trials are necessary to determine the viability of using METTL3 small molecule inhibitors as drugs for treating AD.

## Conclusion and Perspectives

With advances in sequencing technology, methods for detecting m6A have become increasingly sensitive. The latest picoMeRIP technology can identify m6A sites in input RNA amounts as low as 100 pg [96], paving the way for future single-cell m6A mapping in the nervous system. Owing to the similarity between m6Am and m6A modifications, a combination of methods, such as LC‒MS/MS, is necessary to accurately distinguish between them when the effects of m6A on brain development or physiological function are studied. O-GlcNAc modification [97] plays a role in regulating the development of the nervous system and synaptic plasticity through its impact on liquid-liquid phase separation (LLPS). However, the function of RNA methylation in LLPS within the mammalian brain remains unclear. Although our understanding of the function of m6A in brain development is expanding, many areas have not been investigated. For example, METTL3 is a core subunit of the methyltransferase complex that catalyzes the formation of m6A. However, owing to the lack of specific spatiotemporal knockout animal models, the role of METTL3-mediated m6A in mammalian brain development remains unclear. Routine *METTL3* knockout in mice leads to embryonic mortality, highlighting the need for more sophisticated experimental approaches to study this important topic.
